# Morphine with or without Acepromazine in Horses: A Kinematic Evaluation

**DOI:** 10.3390/ani12091193

**Published:** 2022-05-06

**Authors:** F. Javier López-Sanromán, G. Montes Freilich, D. Gómez-Cisneros, J. Izquierdo-Moreno, M. Varela del Arco, G. Manso-Díaz

**Affiliations:** 1 Department of Animal Medicine and Surgery, Faculty of Veterinary Medicine, Universidad Complutense de Madrid, 28040 Madrid, Spain; yoyi83@hotmail.com (G.M.F.); jorge_iz_mo@hotmail.com (J.I.-M.); mtvarela74@gmail.com (M.V.d.A.); gmanso@ucm.es (G.M.-D.); 2 Department of Clinicas and Surgery, Universidad de Panamá, Ciudad de Panama 3366, Panama; david_gomez09@hotmail.com

**Keywords:** gait analysis, accelerometry, acepromazine, morphine, horse

## Abstract

**Simple Summary:**

Morphine is an opioid agonist drug and produces a significant analgesic effect in horses but besides the evidenced analgesic effect, the use of morphine is not routine due to the potential excitatory effects described in the literature. To minimize these effects, neuroleptanalgesia, or the combination of opioids and sedative drugs, is encouraged. Our aim was to describe changes occurring in the locomotor pattern after co-administration of a tranquilizer, acepromazine, and morphine in horses. Six mature horses were used and received four different treatments with saline solution, morphine, acepromazine, or a combination of morphine and acepromazine. A three-dimensional accelerometric device was used to collect data and objectivize those findings moreover the sedative effect of the treatments was also measured. Significant differences were observed when comparing all the treatments in the majority of accelerometric variables, except the regularity of the pattern, some energetic parameters, and tranquilization. An evident counteraction of the effects caused by both morphine and acepromazine was observed. Due to these effects, the possibility of adding acepromazine to an additional analgesic treatment with morphine in the clinical setting ensures the absence of the supplemental instability caused by other sedatives and minimizes the potential opioid excitatory effects.

**Abstract:**

The objective was to demonstrate walking locomotor pattern alterations after co-administration of acepromazine and morphine in horses. Six mature horses receiving four different treatments were used. Treatments consisted of a single dose of saline solution, 0.2 mg/kg bwt of morphine hydrochloride, 0.02 mg/kg bwt of acepromazine maleate, and a combination of 0.2 mg/kg bwt of morphine hydrochloride with 0.02 mg/kg bwt of acepromazine maleate. A three-dimensional accelerometric device was used to collect data. Walking tests were performed 10 min prior to injection, and then at 5, 10, 15, and 20 min after the injection, and then every 10 min for 3 h. Eight variables were calculated including stride kinematic, coordination, and energetic parameters; moreover ground-to-lip distance (GLD), as a tranquilization parameter, was also measured. A significant interaction was observed in all the variables studied but regularity, mediolateral power, the propulsive part of the power, and the GLD. An evident counteraction of the effects caused by both, opioids and phenothiazines, in the gait pattern was observed. The co-administration of acepromazine and morphine could allow a safe opiate administration while minimizing the possible central nervous system (CNS) excitation and reducing potential locomotor adverse effects.

## 1. Introduction

Morphine is the main mu receptor opioid agonist and exerts a significant analgesic effect in humans and small animals making its use of great interest in clinical activity. But besides this analgesic effect in other species, the use of morphine in the equine clinical setting is not routine due to the potential adverse effects described in the literature [1,2]. These undesirable effects include decreased gastrointestinal motility, disorientation, increased locomotor activity, hyper-responsiveness to touch and sound, and the development of ataxia [3]. The increase in locomotor activity is dose-dependent and seems to be less marked in kappa-opioid agonists than that described with mu-agonists [4,5] and is also less common when administered to horses in pain compared with pain-free individuals [4] because the risk of these adverse opioid-mediated reactions seems to be inversely proportional to the extent of the patient’s pain [6]. This dose-dependent locomotor activity with incoordination was first described in 1979 with a marked individual variation in response, setting the median effective value for morphine for increasing locomotion at 0.91 mg/kg, a considerably greater analgesic dose than the one used in the clinical setting [4].

Neuroleptanalgesia refers to an effect produced by the combination of opioids and tranquilizers, or sedative drugs and this combined administration appears to minimize the mentioned excitatory consequences [7]. Alpha-2 adrenergic agonists [8,9] and phenothiazines [7,8,10] have both been used, added to opioids, in order to ameliorate the possible central nervous system (CNS) excitation in horses [11]. It is considered that this locomotor effect results from dopaminergic activity [12] and that the administration of acepromazine, due to phenothiazine blockade of central and peripheral adrenergic and dopaminergic receptors [3], reduces the locomotor response. In fact, the administration of acepromazine, due to its antidopaminergic effect, blocked the locomotor effects of fentanyl and morphine in horses [13].

Recently, using objective gait analysis systems, the administration of a clinically relevant recommended dose of morphine sulfate to conscious healthy horses produced limited but measurable effects on the gait pattern [14]. Therefore, the objective of the present study was to evaluate, using accelerometry, changes occurring in the locomotor pattern after adding acepromazine to morphine while treating non-painful horses. Our hypothesis is that despite the fact that the treated horses showed limited undesirable locomotor effects, the combination with acepromazine will minimize these changes that will be precisely objectified using accelerometry.

## 2. Materials and Methods

### 2.1. Horses

The study was approved by the Complutense University Animal Care and Use Committee. Six adult horses (two geldings and four mares) from our research herd, aged 4–22 years [mean age (±s.d.) of 13.2 ± 8.3 years] and with bodyweights ranging from 418–441 kg [mean (±s.d.) 425.8 ± 10.2 kg] were used in this study. A previous clinical examination was performed on all horses to ensure they were sound and not lame.

### 2.2. Treatments

Each animal was used as its own control, and 4 treatments were administered in a random order to each horse using an online random choice generator: 10 mL of sodium chloride (0.9%) solution (Solución salina fisiológica 0.9% B/BRAUN Medical S.A., Spain) as a control; 0.2 mg/kg bwt of morphine hydrochloride (MOR) (Morfina Braun 20 mg/mL, B/BRAUN Medical S.A., Barcelona, Spain) diluted in sodium chloride solution to a volume of 10 mL; 0.02 mg/kg bwt of acepromazine maleate (ACE) (Equipromacina, 5 mg/mL, Labiana Life Sciences S.A., Tarrasa, Spain) diluted in sodium chloride solution to a volume of 10 mL; a combination of 0.2 mg/kg bwt of morphine hydrochloride with 0,02 mg/kg bwt of acepromazine maleate (MOR + ACE) also diluted in sodium chloride solution to a volume of 10 mL. Each treatment was administered via an intravenous (IV) 16-gauge catheter (Surflash, Terumo Europe N.V., Leuven, Belgium), inserted into the left jugular vein; the catheter was immediately removed after the treatment administration. All drugs were administered at a minimum interval of 14 days.

### 2.3. Data Acquisition

Data were acquired using a portable gait analyzer (Equimetrix, Centaure Metrix, France) including a data logger, an acceleration sensor with three accelerometers measuring along the lateral, longitudinal, and dorsoventral axes of the horse, and a specific scientific software program (Equimetrix-Centaure 3D Matlab 5, The MathWorks Inc., Portola Valley, CA, USA). The recorder collected data continuously at a sampling rate of 100 Hz while the horse was walking. After finishing the test, data were transferred to a computer. Positive values corresponded to acceleration signals in the dorsal, cranial, and left directions. The data logger was maintained in a leather pocket attached to an elastic girth placed on the thorax and connected to the sensor.

### 2.4. Experimental Procedure

All measurements were undertaken in the same quiet environment placing horses in stocks between each of the accelerometric recordings. During the 190 min, a total of thirty-nine walking trials involving an accelerometric gait assessment were performed. For this and with the accelerometer sensor in position, the horse was walked, at its preferred speed along a 50 m runway. Only the walking away from the stables was considered because the horses always walked faster toward the stables. Before starting the test, the horse was instrumented by fixing the triaxial accelerometer to the skin in the most dorsal aspect of the sacral region using double-adhesive tape. Ten minutes before injection of the treatment, the horse was walked three times over a 50 m distance across the track to register baseline accelerometric recordings. Each horse was then injected with one of the studied treatments (0 min), and recordings were repeated at 5, 10, 15, and 20 min after the treatment (single measurement) and then every 10 min until reaching 3 h after injection of the test treatment (two measurements).

### 2.5. Accelerometric Variables

Reproducibility and validation of the accelerometric measurements with the triaxial accelerometric device (Equimetrix, Centaure Metrix, Évry-Courcouronnes, France) used in the present study have been described [15,16,17]. All stride kinematic, coordination and energetic variables evaluated have also been described [16,18,19,20,21] including the following.

Stride kinematic variables such as speed (S; m/s), stride frequency (SF; cycles/s or Hz), and stride length (SL; m). Regularity (REG; dimensionless) as a coordination variable to assess the stride to stride acceleration pattern variability. Additionally, energetic variables such as dorsoventral power (DVP; W/kg); propulsive power (PP; W/kg); mediolateral power (MLP; W/kg); and total power (TP; W/kg), were defined as the sum of all three powers. To calculate the redistribution of the power, the propulsive, mediolateral, and dorsoventral components as a percentage of TP (%PP, %MLP and %DVP respectively) were calculated by dividing the power components by the TP. Finally, the force of acceleration (FA; N/kg) was also calculated by dividing the TP of accelerations by speed in order to avoid potential variations due to different speeds.

All the accelerometric variables studied were calculated every other second at twenty-one different time instants of stabilized movement, starting in the 5th second after initiating the recording process to finish on the twenty-five second. Thus, non-stabilized walking at the beginning and end of the test was not considered. The final value at each time point was calculated as the mean of all the measured values, 63 values for the baseline (−10 min), 21 values at 5, 10, 15, and 20 min, and 42 values at 30, 40, 50, 60, 70, 80, 90, 100, 110, 120, 130, 140, 150, 160, 170, and 180 min after the injection of the treatment.

### 2.6. Tranquilization Assessment

Ground-to-lip distance (GLD) was used for the degree of tranquilization assessment and was measured at each study point before each first walking test. The lowering of the head was measured by determining the position of the nose related to a cm-scale marked in a sidebar of the stock where horses were kept between trials.

### 2.7. Statistical Data Analysis

Data analyses were performed using a computer SAS 9.4 software for Windows (SAS Institute Inc., Carry, NC, USA). Data were organized and summarized as percentage ± SD values relative to baseline measurements. Power components of the total power were expressed by the percentage of TP. First, a two-way analysis of variance (ANOVA) with an inter-subject factor was performed. In case of a significant interaction, a one-way ANOVA was also carried out comparing groups for each time point with the Duncan’s multiple range post hoc test. Additionally, repeated-measures one-way ANOVA for each treatment to find differences between time points using a Dunnett correction test was accomplished. *p* < 0.05 was considered statistically significant.

## 3. Results

All the horses involved in this project completed the study and all the data were included in the statistical analyses. No significant differences were observed between control values at any time. A statistically significant interaction was observed in all the accelerometric variables studied except for REG, MLP, and %PP and also for GLD as a tranquilization parameter. Parameter values at each time point and statistical significance are presented in Table 1, Table 2, Table 3, Table 4, Table 5 and Table 6.

### 3.1. Stride Kinematic Variables

**Figure 1:**
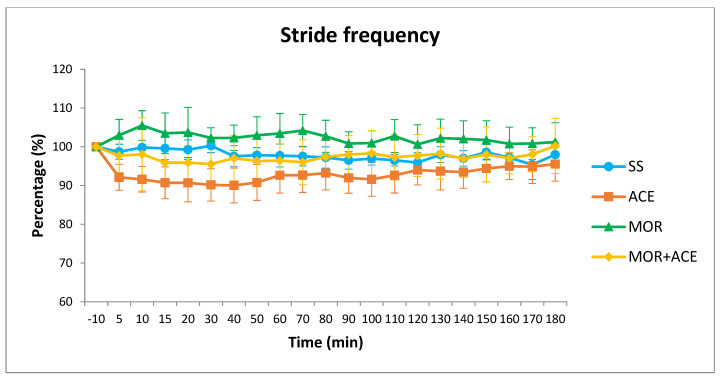
Stride frequency values (% ± SD) at baseline (−10 min) and at 5, 10, 15, 20, and then every 10 min (total period of 3 h) after IV injection (at 0 min) of 10 mL of sodium chloride (0.9%) (SS), 0.2 mg/kg of morphine hydrochloride solution diluted in sodium chloride (0.9%) to a volume of 10 mL (MOR), 0.02 mg/kg of acepromazine maleate diluted in sodium chloride (0.9%) to a volume of 10 mL (ACE), or a combination solution of 0.2 mg/kg of morphine hydrochloride and 0.02 mg/kg of acepromazine maleate diluted in sodium chloride (0.9%) to a volume of 10 mL (MOR + ACE).

Comparing all four treatments, significant differences in speed (*p* < 0.0001), SF (*p* < 0.0001) and SL (*p* = 0.007) were detected. These differences lasted 140 min for speed and 160 min for SF while differences in SL were less consistent appearing at different time points during the 3-h studying period. Compared to baseline values, in the case of speed, no changes were observed in the MOR group while significant reductions for 180 min and 90 min were observed in the ACE and MOR + ACE groups respectively. For SF, significant reductions for 180 min were also observed in the ACE group while in the MOR group no differences were observed. In the combination group, differences were only observed 10 min after the injection of the combination (Figure 1). Finally, for SL values, differences were observed in the MOR group for 80 min and again 110 min after treatment. In the ACE group differences were observed at 20, 30, and 40 min and again at 70 and 100 min after the phenothiazine administration. After the combination, differences were only observed at 10, 15, 20, and 30 min.

### 3.2. Coordination Variable

No differences among treatments were observed for REG values with a *p* = 0.5074 when comparing all four treatments.

### 3.3. Energetic Variables

Regarding power values and comparing all four treatments, significant differences in DVP (*p* = 0.0002), PP (*p* < 0.0001) and TP (*p* = 0.0005) were detected. These differences started 10 min after treatment and lasted 50 min for DVP. For PP lasted 50 min while differences in TP lasted 40 min. Compared to baseline values, in the case of DVP, no changes were observed in the MOR group while significant reductions for 180 min were observed in the ACE group and only differences 10 min after treatment were observed in the MOR + ACE group. For PP, significant reductions for 150 and then at 170 min were observed in the ACE group while in the MOR and combination groups, differences were observed at 5 and then from 15 to 40 min after treatment and from 10 to 70 min after injection respectively. Finally, for TP values, significant reductions for 150 min were observed in the ACE group while in the MOR group no differences were observed. In the MOR + ACE group, differences were only observed from 10 to 30 min after the injection of the combination.

**Figure 2:**
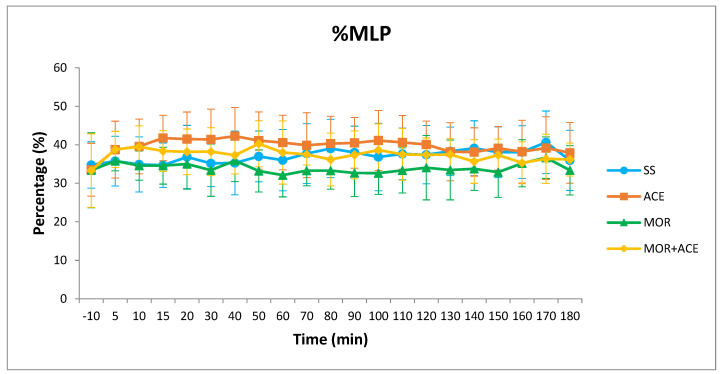
Mediolateral part of the power values (% ± SD) at baseline (−10 min) and at 5, 10, 15, 20, and then every 10 min (total period of 3 h) after IV injection (at 0 min) of 10 mL of sodium chloride (0.9%) (SS), 0.2 mg/kg of morphine hydrochloride solution diluted in sodium chloride (0.9%) to a volume of 10 mL (MOR), 0.02 mg/kg of acepromazine maleate diluted in sodium chloride (0.9%) to a volume of 10 mL (ACE), or a combination solution of 0.2 mg/kg of morphine hydrochloride and 0.02 mg/kg of acepromazine maleate diluted in sodium chloride (0.9%) to a volume of 10 mL (MOR + ACE).

In reference to force and the parts of the TP values, significant differences in FA (*p* = 0.0195), %DVP (*p* < 0.0077) and %MLP (*p* = 0.0054) were detected. These differences were less consistent for these parameters appearing only in minutes 30 and 15 for FA and %MLP respectively and at minutes 10, 15, 20 40, 60, and 70 for %DVP values. Compared to baseline values, in the case of FA, no changes were observed in the MOR and MOR + ACE groups while significant reductions for 130 min were observed in the ACE group. For %DVP, no changes were observed in the MOR group while significant reductions for 180 min were observed in the ACE group and only differences 50 min after treatment were observed in the MOR + ACE group. Finally, for %MLP values, significant reductions from minute 10 to 130 and again 160 min after treatment were observed in the ACE group while in the MOR group no differences were observed. In the MOR + ACE group, differences were only observed for 10 min after the injection of the combination (Figure 2).

### 3.4. Tranquilization Variable

No differences, when comparing all four treatments, were observed for GLD values (*p* = 0.1523).

## 4. Discussion

We found that possibly due to the limited excitatory effect of morphine at the dose studied in the present study (0.2 mg/kg), the effects of adding acepromazine were only recognizable in a limited number of accelerometric variables but amelioration of the acepromazine effects was observed when combined with morphine, being evident a minimization of the normal alterations that acepromazine causes in the gait pattern of horses [22].

It has been described that the reasons for a co-administration of a sedative drug with opioids are the lack of predictability, the inability to produce desired effects, and the development of side effects, especially excitement and ataxia at higher doses [10]. The purpose of our study was to demonstrate changes occurring in the locomotor pattern after co-administration of acepromazine and morphine while treating non-painful horses.

All the phenothiazine derivates, including acepromazine, appear to produce part of their central effects by blocking brain dopaminergic receptors [23] although the activation of these dopaminergic pathways is a cause of opioid-induced spontaneous locomotor activity is controversially discussed [1,10]. Additionally, the blockade of this opioid-induced dopamine release by phenothiazine tranquilizers is considered a rational alternative to inhibiting the excitement and producing more predictable calming and pain relief effects [3,24].

In the present study, we wanted to use doses used in clinical settings which are substantially less than those used in some experimental studies because, even with low doses, the excitatory effects of opioids are unpredictable and discrepancies exist between the analgesic properties and potential side effects [11].

In the case of the studied kinematic variables, when compared to the control treatment, the combination of morphine and acepromazine produced, a reduction in speed lasting for 40 min after administration. Nevertheless, acepromazine produced a longer reduction in speed, lasting 100 min more, while morphine alone did not produce significant changes in velocity. Velocity is the product of SF and SL and these two parameters change in a linear fashion with velocity so that faster velocities are achieved by increasing SF and SL almost proportionally (44 and 56%) [25]. The shorter reduction in speed produced after the administration of the combination was mainly due to the increase in SF produced after the injection of morphine that, despite not being globally significant, produced an increase in SF values in all time points. This increase somewhat countered the speed decrease caused after the administration of acepromazine in the first measured time periods. The SL values, on the other hand, were reduced significantly in the morphine group but were not sufficient to cause a reduction in speed in that group of treatment. At this opioid dose, the effects of morphine when combined with acepromazine were comparable to the changes produced with morphine alone but with significant changes for a shorter period, lasting only 30 min. Globally, comparing the effects of the combination to the administration of saline solution alone, we found that the administration of the combination of morphine and acepromazine produced inconsistent and shorter effects in the kinematic variables resembling more the ones produced by the administration of acepromazine alone and, therefore, far from the effects of increased locomotor activity produced by morphine. As we can see in Figure 1, SF values are a good example of the mitigation that the co-administration of acepromazine produces in the potential increased locomotor activity caused by opioids.

In the case of REG (i.e., stride-to-stride variability), which is an accelerometric-specific variable, no differences among treatments were observed. Neither morphine nor acepromazine administration produced significant differences in REG values and, as expected, neither did the combination of both drugs. REG has been described as a very sensitive parameter to detect and quantify uncoordinated movements at the walk. The administration of xylazine (0.5 mg/kg), detomidine (0.01 mg/kg), and romifidine (0.04 mg/kg) IV always produces a significant reduction in REG values in horses [26]. Combining detomidine and butorphanol, a synthetic agonist-antagonist opioid, produced a greater but not significant reduction in REG results measured 5 min after the injection but, from that moment onwards, the decreases in REG values were less severe in the detomidine and butorphanol combination group [27]. On the other hand, in our study, the combination of acepromazine and morphine did not produce a summatory or synergic effect on this coordination parameter with equivalent values (i.e., no significant changes in REG values) after the combination injection. This could be an advantage of using phenothiazines instead of alpha-2 adrenergic agonists for neuroleptanalgesia in horses being the use of acepromazine is a safe measure in terms of stability in horses with ataxia or incoordination when additional analgesic treatment is needed.

In addition, the administration of a combination of acepromazine and morphine produced only mild effects in power values being the most obvious in the PP values. TP and FA values suffered only isolated time point alterations during the first 30 min after the administration of the combination. On the other hand, the administration of acepromazine alone produced significant reductions in the energetic variables, even at lower doses (0.01 mg/kg) and specifically in MLP, TP, and FA values [27]. Those effects were probably due to the tranquilization effect of the phenothiazine and because an evident relation between power and speed has been described [28]. In this study, the addition of morphine produced a minimization of the mentioned power results described after acepromazine injection. Again, we found that the results in power values after the combination administration were very close to the ones obtained after the administration of saline solution alone showing up, therefore, a very safe treatment due to the obvious blockade of the effects caused by acepromazine in the locomotor patterns.

Moreover, 3D accelerometry allows determining the redistribution of power in the three axial directions. In our case, the three-axial distribution of the power values was globally maintained with around 30% for the DVP, 35% for the PP, and 35% for the MLP approximately after the administration of the combination. In the case of %PP, the administration of morphine produced a significant decrease of the values lasting 20 min when compared to baseline values. These reduced values could be the result of changes in weight-bearing and symmetry produced by the excitatory effect of morphine [14,29] and were completely minimized by the administration of the combination. In the redistribution of the power values, the combination again produced effects similar to those caused by the administration of the saline solution. It is important to mention the effects of adding acepromazine in the %MLP values (Figure 2). As described for other sedative drugs, especially alpha-2 agonist drugs, sedation causes a redistribution of the three-axial power with an increase in the %MLP values mainly at the expense of %DVP values [15]. This effect has been attributed to the uncoordinated waddling and rolling gait in sedated horses [21] and the addition of morphine again minimized the redistribution of forces maintaining stable values for the %MLP from minute 15 onwards after the administration of the combination.

Finally, no GLD reduction effects were observed after the administration of the combination of acepromazine and morphine. Morphine injection also did not produce sedative effects while a dose of 0.02 mg/kg did produce long-lasting sedative effects of up to 160 min. Again, we observe in our study that, in some way, the effects of morphine and those of acepromazine are antagonistic and, in this case, morphine completely abolished the effect produced by the phenothiazine administration. We considered these antagonistic effects of special importance in the case of tranquilization. Several studies describe better sedation promoted with alpha-2 adrenergic agonists compared to phenothiazines when combined with opioids, certifying a synergistic effect of both drug groups [30,31]. In our study, the abolition of sedative effects could make a big difference in ataxic horses. Both alpha-2 adrenergic agonists and phenothiazines are used to produce sedation in horses but the use of alpha-2 produces more sedative effects, and this additional sedation could cause greater instability which, in certain clinical activities, could be the cause of potential clinical negative consequences.

Besides the limited number of animals used in the present study, another limitation observed was the high frequency of the accelerometric measurements that could maybe interfere with the tranquilization effect of acepromazine adding certain disturbances to the animals preventing a correct tranquilization. This limitation is inherent to the experimental protocol being especially evident in the first four intervals. Another important limitation is derived from the tranquilization assessment method used. Although GLD has been used to measure the sedative effect of different drugs, including the phenothiazine tranquilizers [32] is an assessment method more frequently used to evaluate the sedative effect of certain drugs, especially alpha-2 adrenergic agonists in which an important myorelaxation effect is evident. Maybe the use of other methods to evaluate the response to other stimuli would have provided additional important information.

## 5. Conclusions

The possibility of adding acepromazine to another analgesic treatment in an ataxic horse or during the postoperative treatment of an orthopedic surgical case ensures the absence of the supplemental instability caused by other sedatives used in the daily clinical setting.

In conclusion, because of the possible individual variation in response to opioids and the lack of predictability, the co-administration of acepromazine and morphine could provide the desired analgesic treatment while minimizing the possible CNS excitation and could be a good choice in providing balanced analgesia for perioperative pain-reducing potential excitatory locomotor adverse effects and being a safe combination for horses with incoordination or ataxia.

## Figures and Tables

**Table 1:** Values at walk of stride kinematic variables at −10, 5, 10, 15, 20, and then every 10 min (total period of 3 h) after IV injection (at 0 min) of 10 mL of sodium chloride (0.9%) solution (SS), 0.2 mg/kg of morphine hydrochloride solution diluted in sodium chloride (0.9%) to a volume of 10 mL (MOR), 0.02 mg/kg of acepromazine maleate diluted in sodium chloride (0.9%) to a volume of 10 mL (ACE), or a combination solution of 0.2 mg/kg of morphine hydrochloride and 0.02 mg/kg of acepromazine maleate diluted in sodium chloride (0.9%) to a volume of 10 mL (MOR + ACE).

VARIABLE		S				SF				SL			
TREATMENT		SS	MOR	ACE	MOR + ACE	SS	MOR	ACE	MOR + ACE	SS	MOR	ACE	MOR + ACE
Baseline		100 ± 0	100 ± 0	100 ± 0	100 ± 0	100 ± 0	100 ± 0	100 ± 0	100 ± 0	100 ± 0	100 ± 0	100 ± 0	100 ± 0
**Time after administration of treatment (min)**	5	97.98 ± 1.94 ^‡^	98.09 ± 4.54 ^‡^	90.64 ± 5.54 ^#,†,^*	94.56 ± 2.77 *	98.7 ± 1.96 ^‡^	103 ± 4.06 ^§,‡^	92.12 ± 3.36 ^#,§,†,^*	97.71 ± 4.36 ^‡,†^	99.33 ± 2.26	95.27 ± 3 *	98.39 ± 3.91	96.83 ± 3.13
	10	97.71 ± 3.93 ^§,‡^	100.4 ± 3.85 ^§,‡^	89.1 ± 5.19 ^#,†,^*	91.73 ± 2.24 ^#,†,^*	99.83 ± 0.74 ^‡^	105.5 ± 3.83 ^§,‡,^*	91.59 ± 3.3 ^#,§,†,^*	98.06 ± 9.43 ^‡,†^	97.88 ± 3.76	95.17 ± 1.38 *	97.31 ± 4.03	95.22 ± 4.24 *
	15	100.12 ± 4.98 ^§,‡^	95.96 ± 6.6 ^‡^	87.63 ± 3.95 ^#,†,^*	91.57 ± 3.19 ^#,^*	99.59 ± 2.9 ^‡^	103.49 ± 5.22 ^§,‡^	90.71 ± 4.11 ^#,§,†,^*	95.91 ± 4.47 ^‡,†^	100.6 ± 4.65	92.84 ± 6.42 *	96.76 ± 4.42	95.54 ± 1.73 *
	20	101.69 ± 5.32 ^§,‡^	96.3 ± 5.37 ^‡^	86.83 ± 5.13 ^#,†,^*	91.49 ± 4.44 ^#,^*	99.25 ± 2.52 ^‡,†^	103.7 ± 6.48 ^#,§,‡^	90.68 ± 4.9 ^#,§,†,^*	95.93 ± 4.27 ^‡,†^	102.46 ± 4.22 ^§,‡,†^	93.08 ± 5.95 ^#,^*	95.8 ± 2.3 ^#,^*	95.37 ± 2.67 ^#,^*
	30	102.77 ± 6.93 ^§,‡^	98.05 ± 1.9 ^‡^	85.74 ± 6.94 ^#,†,^*	91.46 ± 4.36 ^#,^*	100.28 ± 1.79 ^§,‡^	102.29 ± 2.62 ^§,‡^	90.16 ± 4.18 ^#,§,†,^*	95.55 ± 5.65 ^#,‡,†^	102.51 ± 6.78	95.94 ± 3.28 *	95.04 ± 3.68 *	95.79 ± 2.04 *
	40	101.19 ± 3.62 ^§,‡^	97.08 ± 3 ^‡^	86.07 ± 5.8 ^§,#,†,^*	93.55 ± 4.72 ^#,‡,^*	97.55 ± 2.71 ^‡,†^	102.3 ± 3.27 ^#,§,‡^	90.02 ± 4.48 ^#,†,^*	97.06 ± 5.81 ^‡,†^	103.79 ± 4.03 ^§,‡,†^	94.97 ± 3.32 ^#,^*	95.57 ± 2.65 ^#,^*	96.42 ± 2.22 ^#^
	50	99.58 ± 4.79 ^‡^	97.9 ± 4.67 ^‡^	87.32 ± 3.51 ^#,†,^*	92.9 ± 6.62 *	97.84 ± 1.94 ^‡,†^	102.97 ± 4.78 ^#,§,‡^	90.8 ± 4.63 ^#,§,†,^*	96.33 ± 6.67 ^‡,†^	101.86 ± 4.87 ^§,‡,†^	95.14 ± 3.93 ^#,^*	96.28 ± 2.37 ^#^	96.49 ± 3.57 ^#^
	60	98.52 ± 4.48 ^‡^	98.26 ± 6.01^‡^	89.13 ± 4.12 ^#,†,^*	93.65 ± 6.26 *	97.74 ± 2.95 ^†^	103.46 ± 5.14 ^#^	92.66 ± 4.59 *	96.45 ± 4.32	100.81 ± 3.15	95 ± 4.32 *	96.23 ± 1.48	97.04 ± 3.86
	70	97.78 ± 3.1 ^‡^	99.02 ± 5.43 ^‡^	88.76 ± 6 ^#,†,^*	92.82 ± 4.26 *	97.55 ± 2.49 ^†^	104.21 ± 4.12 ^#,‡^	92.66 ± 4.43 ^†,^*	96.02 ± 5.78	100.24 ± 2.41	95.1 ± 4.95 *	95.76 ± 2.21*	96.77 ± 3.63
	80	99.41 ± 4.62 ^‡^	97.21 ± 4.51 ^‡^	90.42 ± 4.31 ^#,†,^*	94.32 ± 5.64 *	97.24 ± 2.76^†^	102.72 ± 4.13 ^#,§,‡^	93.23 ± 4.37 ^†,^*	97.41 ± 4.91 ^†^	102.27 ± 3.56 ^§,‡,†^	94.7 ± 3.23 ^#,^*	97.05 ± 2.71^#^	96.81 ± 2.43 ^#^
	90	99.12 ± 4 ^‡^	97.5 ± 3.3 ^‡^	88.58 ± 3.87 ^#,§,†,^*	94.58 ± 3.77 ^‡,^*	96.51 ± 2.25 ^‡,†^	100.86 ± 3.03 ^#,‡^	91.98 ± 3.95 ^#,§,†,^*	98 ± 4.92 ^‡^	102.77 ± 3.88 ^§,‡,†^	96.72 ± 3.7 ^#^	96.4 ± 2.98 ^#^	96.6 ± 2.57 ^#^
	100	96.6 ± 2.01 ^‡^	97.46 ± 3.72 ^‡^	87.41 ± 6.19^#,§,†,^*	95.28 ± 3.51 ^‡^	96.96 ± 1.69 ^‡,†^	100.98 ± 3.15 ^#,‡^	91.6 ± 4.37 ^#,§,†,^*	98.32 ± 5.86 ^‡^	99.72 ± 3.3	96.57 ± 3.83	95.4 ± 3.09 *	97.05 ± 4.45
	110	96.18 ± 2.15 ^‡^	98.23 ± 4.17 ^‡^	89.09 ± 5.1 ^#,§,†,^*	95.73 ± 5.3 ^‡^	96.53 ± 1.48 ^†^	102.77 ± 4.26 ^#,§,‡^	92.65 ± 4.58 ^§,†,^*	97.31 ± 5.82 ^‡,†^	99.72 ± 3.06 ^‡,†^	95.63 ± 2.55 ^#,^*	96.17 ± 1.92 ^#^	98.41 ± 2.22
	120	97.65 ± 3.75 ^‡^	98.71 ± 4.68 ^§,‡^	91.28 ± 3.64 ^#,†,^*	95.98 ± 5.13 ^†^	95.92 ± 2.57	100.65 ± 5.04 ^‡^	93.99 ± 3.81 ^†,^*	97.74 ± 5.42	101.88 ± 3.68	98.11 ± 2.83	97.17 ± 2.42	98.23 ± 1.17
	130	99.7 ± 5.04 ^‡^	99.71 ± 4.61 ^§,‡^	90.94 ± 4.8 ^#,†,^*	95.51 ± 5.62 ^†^	97.98 ± 2.03	102.21 ± 4.92 ^‡^	93.7 ± 4.82 ^†,^*	98.23 ± 6.58	101.8 ± 4.94 ^§,‡,†^	97.58 ± 1.33 ^#^	97.12 ± 1.9 ^#^	97.3 ± 3.17 ^#^
	140	97.63 ± 2.88 ^‡^	99.13 ± 4.86 ^‡^	91.26 ± 2.74 ^#,†,^*	95.52 ± 3.4	97.01 ± 2 ^‡,†^	102.06 ± 4.65 ^#,§,‡^	93.47 ± 4.17 ^#,§,†,^*	96.94 ± 4.82 ^‡,†^	100.75 ± 4.38	97.15 ± 2.66	97.75 ± 1.84	98.62 ± 2.77
	150	97.13 ± 1.9 ^‡^	98.48 ± 4.36 ^‡^	92.82 ± 3.07 ^#,†,^*	96.44 ± 7.16	98.61 ± 2 ^‡^	101.76 ± 4.97 ^‡^	94.39 ± 3.46 ^#,†,^*	98.02 ± 7.11	98.54 ± 2.31	96.83 ± 2.75	98.42 ± 1.71	98.39 ± 2.18
	160	97.81 ± 4.39 ^‡^	97.79 ± 4.21 ^‡^	93.69 ± 5.47 ^#,†,^*	95.65 ± 4.26	97.19 ± 2.78	100.78 ± 4.27 ^‡^	95 ± 3.44 ^†,^*	97.03 ± 3.99	100.71 ± 3.56	97.13 ± 4.04	98.65 ± 2.73	98.6 ± 2.95
	170	98.24 ± 5.18 ^‡^	98.05 ± 2.12 ^‡^	93.99 ± 5.06 ^#,†,^*	96.57 ± 3.52	95.38 ± 3.83	100.84 ± 4.06	94.86 ± 4.31 *	98.06 ± 4.53	103.03 ± 3.39 ^§,‡,†^	97.34 ± 3.37 ^#^	99.1 ± 0.9 ^#^	98.55 ± 2.83 ^#^
	180	102.21 ± 5.65 ^‡^	97.9 ± 4.59 ^‡^	93.89 ± 4.14 ^#,†,^*	99.57 ± 6.12	98 ± 3	101.2 ± 5.02	95.53 ± 4.41 *	100.22 ± 7.13	104.32 ± 4.42 ^§,‡,†^	96.8 ± 3.05 ^#^	98.32 ± 1.43 ^#^	99.43 ± 2.65 ^#^

S = speed, SF = stride frequency, SL = stride length. All variables are expressed as a mean percentage ± SD, relative to baseline values. ^#^ For a given variable, the value is significantly (*p* < 0.05) different from the saline solution at that time point. ^§^ For a given variable, the value is significantly (*p* < 0.05) different from the combination of morphine and acepromazine value at that time point. ^‡^ For a given variable, the value is significantly (*p* < 0.05) different from the acepromazine value at that time point. ^†^ For a given variable, the value is significantly (*p* < 0.05) different from the morphine value at that time point. * For a given variable, the value is significantly (*p* < 0.05) different from the baseline value at that time point.

**Table 2:** Values at walk of coordination variable at −10, 5, 10, 15, 20, and then every 10 min (total period of 3 h) after IV injection (at 0 min) of 10 mL of sodium chloride (0.9%) solution (SS), 0.2 mg/kg of morphine hydrochloride solution diluted in sodium chloride (0.9%) to a volume of 10 mL (MOR), 0.02 mg/kg of acepromazine maleate diluted in sodium chloride (0.9%) to a volume of 10 mL (ACE), or a combination solution of 0.2 mg/kg of morphine hydrochloride and 0.02 mg/kg of acepromazine maleate diluted in sodium chloride (0.9%) to a volume of 10 mL (MOR + ACE).

VARIABLE		REG			
TREATMENT		SS	MOR	ACE	MOR + ACE
Baseline		100 ± 0	100 ± 0	100 ± 0	100 ± 0
**Time after administration of treatment (min)**	5	94.45 ± 7.86	92.63 ± 15.55	85.88 ± 6.24	93.3 ± 10.72
	10	100.85 ± 10.27	97.95 ± 19.1	86.93 ± 12.85	88.08 ± 9.17
	15	96.86 ± 12.5	94.57 ± 18.93	82.64 ± 8.95	93.64 ± 14.8
	20	101.9 ± 6.57	98.32 ± 16.09	87.45 ± 11.24	94.87 ± 17.54
	30	98.09 ± 11.06	94.1 ± 5.54	82.64 ± 13.92	95.72 ± 10.7
	40	98.07 ± 9.98	100.21 ± 12.54	89.27 ± 6.76	96.33 ± 13.13
	50	94.99 ± 15.47	97.96 ± 6.68	80.55 ± 11.68	94.58 ± 13.49
	60	94.7 ± 7.93	100.13 ± 11.36	90.5 ± 10.2	87.16 ± 21.78
	70	96.5 ± 7.55	97.87 ± 8.25	87.03 ± 10.94	95.21 ± 13.97
	80	95.19 ± 13.64	98.51 ± 11.2	91.52 ± 10.5	92.91 ± 21.57
	90	99.06 ± 5.32	106.99 ± 11.71	90.65 ± 13.38	98.53 ± 23.47
	100	92.47 ± 8.72	102.92 ± 11.11	90.3 ± 11.59	100.24 ± 20.61
	110	96.52 ± 6.11	95.09 ± 12.82	83.26 ± 13.8	102 ± 14.53
	120	90.13 ± 15.6	99.05 ± 5.77	88.57 ± 7.81	97.88 ± 14.68
	130	95.78 ± 8.77	102.93 ± 10.91	90.7 ± 6.02	100.84 ± 13.21
	140	93.78 ± 8.9	101.31 ± 11.97	87.59 ± 6.81	98.34 ± 9.12
	150	96.83 ± 6.93	96.47 ± 8.05	89.17 ± 7.59	101.75 ± 11.23
	160	97.16 ± 16.64	93.77 ± 11.54	91.63 ± 13.53	100.64 ± 9.01
	170	95.56 ± 9.18	98.25 ± 16.75	90.8 ± 10.69	102.17 ± 13.36
	180	104.78 ± 8.11	99.87 ± 10.87	94.89 ± 10.2	103.09 ± 13.16

REG = regularity. All variables are expressed as a mean percentage ± SD, relative to baseline values.

**Table 3:** Values at walk of energetic variables at −10, 5, 10, 15, 20, and then every 10 min (total period of 3 h) after IV injection (at 0 min) of 10 mL of sodium chloride (0.9%) solution (SS), 0.2 mg/kg of morphine hydrochloride solution diluted in sodium chloride (0.9%) to a volume of 10 mL (MOR), 0.02 mg/kg of acepromazine maleate diluted in sodium chloride (0.9%) to a volume of 10 mL (ACE), or a combination solution of 0.2 mg/kg of morphine hydrochloride and 0.02 mg/kg of acepromazine maleate diluted in sodium chloride (0.9%) to a volume of 10 mL (MOR + ACE).

VARIABLE		DVP				PP				MLP			
TREATMENT		SS	MOR	ACE	MOR + ACE	SS	MOR	ACE	MOR + ACE	SS	MOR	ACE	MOR + ACE
Baseline		100 ± 0	100 ± 0	100 ± 0	100 ± 0	100 ± 0	100 ± 0	100 ± 0	100 ± 0	100 ± 0	100 ± 0	100 ± 0	100 ± 0
**Time after** **administration of treatment (min)**	5	89.97 ± 6.52	103.17 ± 44.75	59.6 ± 11.54 *	84.06 ± 22.11	96.45 ± 5.18 ^‡,†^	79.29 ± 17.65 ^#,^*	70.37 ± 12.11 ^#,^*	84.79 ± 10.21	97.6 ± 7.98	100.82 ± 32.83	82.96 ± 19.78	111.11 ± 32.77
	10	90.78 ± 12.09 ^‡^	118.56 ± 39.49 ^§,‡^	57.37 ± 14.5 ^#,†,^*	67.5 ± 17.08 ^†,^*	96.45 ± 8.73 ^§,‡^	87.05 ± 18.44 ^‡^	70.28 ± 7.47 ^#,†,^*	75.53 ± 10.65 *	95.2 ± 19.48	109.91 ± 36.84	83.92 ± 18.38	94.44 ± 13.61
	15	94.87 ± 26.28 ^‡^	107.89 ± 42.4 ^§,‡^	51.94 ± 10.99 ^#,†,^*	72.63 ± 25.08 ^†^	92.36 ± 11.92 ^§,‡^	83.95 ± 19.26 ^‡,^*	63.5 ± 5.76 ^#,§,†,^*	79.12 ± 17.59 ^‡,^*	93.45 ± 19.99	100.82 ± 32.83	83.92 ± 18.38	93.33 ± 8.16
	20	106.63 ± 42.42 ^‡^	104.08 ± 28.74 ^§,‡^	50.88 ± 13.46 ^#,†,^*	74.82 ± 18.27 ^†^	108.83 ± 37.02 ^§,‡,†^	79.58 ± 19.03 ^#,^*	65.49 ± 10.75 ^#,^*	78.68 ± 14.04 *	100.14 ± 22.3	97.79 ± 26	82.96 ± 19.78	95.56 ± 15.15
	30	102.33 ± 13.22 ^§^	95.57 ± 26.47 ^‡^	52.51 ± 16.21 ^#,†,^*	70.02 ± 24.35 *	102.78 ± 11.07 ^§,‡,†^	83.45 ± 12.46 ^#,‡,^*	67.29 ± 11.5 ^#,†,^*	75.18 ± 16.01 ^#^	104.02 ± 10.19	88.7 ± 11.48	83.92 ± 18.38	88.89 ± 13.61
	40	93.12 ± 15.84 ^‡^	94.74 ± 26.44 ^‡^	50 ± 12.43 ^#,§,†^,*	79.84 ± 30.82 ^‡^	94.15 ± 12.83 ^‡^	81.78 ± 12.89 ^‡,^*	62.85 ± 9.42 ^#,†,^*	79.36 ± 18.04 *	96.96 ± 19.89	100.82 ± 32.83	82.96 ± 19.78	92.22 ± 8.61
	50	91.65 ± 18.24 ^‡^	98.28 ± 27.03 ^‡^	56.14 ± 17.01 ^#,†,^*	72.67 ± 31.03	91.37 ± 12.82 ^‡^	87 ± 14.75 ^‡^	66.44 ± 9.34 ^#,†,^*	80.83 ± 18.47 *	99.33 ± 10.89	91.73 ± 14.24	84.92 ± 17.34	103.89 ± 32.69
	60	92.38 ± 22.61	107.37 ± 32.86	63.74 ± 18.64 *	76.79 ± 20.52	92.69 ± 10.84	90.17 ± 20.03	75.74 ± 15.62 *	81.36 ± 19.24 *	96.96 ± 19.89	91.73 ± 14.24	93.47 ± 14.71	97.22 ± 22.15
	70	85.43 ± 15.8	113.15 ± 51.3	58.35 ± 17.59 *	78.62 ± 35.35	88.5 ± 16.38	90.9 ± 16.07	73.72 ± 15.18 *	80.79 ± 14.32 *	96.96 ± 7.06	100.82 ± 26.09	85.82 ± 16.22	94.44 ± 17.21
	80	88.63 ± 14.74	97.36 ± 32.62	61.67 ± 19.98 *	85.59 ± 29.16	90.43 ± 18.98	87.92 ± 16.93	76.85 ± 17.21 *	86.68 ± 20.6	105.78 ± 7.44	91.73 ± 14.24	93.44 ± 26.84	95 ± 7.82
	90	82.26 ± 11.13 ^‡^	95.68 ± 29.33 ^‡^	55.93 ± 11.68 ^#,§,†,^*	85.89 ± 27.25 ^‡^	86.56 ± 14.04	88.21 ± 9.26	71.01 ± 8.35 *	87.21 ± 17.96	95.98 ± 8.72	88.7 ± 11.48	86.77 ± 15.56	103.33 ± 24.04
	100	81.57 ± 11.02	95.67 ± 30.86 ^‡^	56.55 ± 15.24 ^†,^*	80.42 ± 25.06	88.55 ± 11.54	88.9 ± 12.19	70.99 ± 15.03 *	89.83 ± 20.09	93.24 ± 17.23	88.7 ± 11.48	87.73 ± 15.24	108.33 ± 29.34
	110	76.79 ± 13.54	105.77 ± 29.35 ^‡^	61.29 ± 19.36 ^†,^*	82.74 ± 28.44	83.89 ± 11.03	93.53 ± 17.02	72.66 ± 11.57 *	90.71 ± 21.51	90.51 ± 11.93	101.7 ± 32.19	90.58 ± 16.35	103.33 ± 24.04
	120	71.43 ± 15.27	94.28 ± 19.25	63.7 ± 13.15 *	82.93 ± 27.62	85.29 ± 10.38	91.72 ± 14.69	77.61 ± 9.77 *	91.47 ± 22.49	87.77 ± 13.94	95.84 ± 11.91	93.55 ± 6.86	105 ± 31.53
	130	86.68 ± 12.79	105.06 ± 24.17	67.6 ± 19.89 *	80.25 ± 28.53	90.37 ± 10.89	96.13 ± 15.12	82.06 ± 15.16 *	92.51 ± 22.31	102.84 ± 8.07	104.73 ± 39.26	90.57 ± 16.27	103.33 ± 24.04
	140	81.79 ± 14.54 ^†^	106.3 ± 27.12 ^#,§,‡^	67.15 ± 11.48 ^†,^*	74.36 ± 22.75 ^†^	87.01 ± 10.4	93.41 ± 18.13	82.06 ± 13.86 *	88.56 ± 21.65	99.54 ± 14.4	102.57 ± 24.64	92.47 ± 18.42	90 ± 11.55
	150	86 ± 14.61	98.42 ± 23.29	69.09 ± 8.44 *	85.22 ± 36.19	92.42 ± 9.67	92.68 ± 16.31	81.18 ± 11.67 *	95.18 ± 27.45	100.21 ± 9.56	93.48 ± 12.94	97.58 ± 20.24	105 ± 16.83
	160	82.06 ± 15.6	92.68 ± 20.78	73.91 ± 18.58 *	78.8 ± 24.75	93.32 ± 12.28	88.94 ± 14.66	86.3 ± 16	92.05 ± 21.36	99.9 ± 3.53	100.82 ± 26.09	99.73 ± 29.8	92.78 ± 11.63
	170	75.99 ± 19.16	92.7 ± 22.31	75.24 ± 17.92 *	82.94 ± 26.66	84.19 ± 18	90.5 ± 17.89	84.28 ± 15.69	93.05 ± 18.93	99.9 ± 6.33	109.91 ± 36.84	102.14 ± 25.61	111.11 ± 32.77
	180	92.72 ± 25.42	92.12 ± 27.44	75.53 ± 15.33 *	95.43 ± 43.46	98.86 ± 6.55	91.89 ± 19.85	86.79 ± 15.22	99.67 ± 21.09	98.92 ± 8.56	91.33 ± 10.57	100.63 ± 27.98	94.44 ± 13.61

DVP = dorsoventral power, PP = propulsion power, MLP = mediolateral power. All variables are expressed as a mean percentage ± SD, relative to baseline values. ^#^ For a given variable, the value is significantly (*p* < 0.05) different from saline solution at that time point. ^§^ For a given variable, the value is significantly (*p* < 0.05) different from the combination of morphine and acepromazine value at that time point. ^‡^ For a given variable, the value is significantly (*p* < 0.05) different from the acepromazine value at that time point. ^†^ For a given variable, the value is significantly (*p* < 0.05) different from the morphine value at that time point. * For a given variable, the value is significantly (*p* < 0.05) different from the baseline value at that time point.

**Table 4:** Values at walk of energetic variables at −10, 5, 10, 15, 20, and then every 10 min (total period of 3 h) after IV injection (at 0 min) of 10 mL of sodium chloride (0.9%) solution (SS), 0.2 mg/kg of morphine hydrochloride solution diluted in sodium chloride (0.9%) to a volume of 10 mL (MOR), 0.02 mg/kg of acepromazine maleate diluted in sodium chloride (0.9%) to a volume of 10 mL (ACE), or a combination solution of 0.2 mg/kg of morphine hydrochloride and 0.02 mg/kg of acepromazine maleate diluted in sodium chloride (0.9%) to a volume of 10 mL (MOR + ACE).

VARIABLE		TP				FA			
TREATMENT		SS	MOR	ACE	MOR + ACE	SS	MOR	ACE	MOR + ACE
Baseline		100 ± 0	100 ± 0	100 ± 0	100 ± 0	100 ± 0	100 ± 0	100 ± 0	100 ± 0
**Time after** **administration of treatment (min)**	5	94.75 ± 5.55 ^‡^	89.8 ± 21.59 ^‡^	70.38 ± 10.06 ^§,†,^*	109.44 ± 29.09 ^#,‡^	96.77 ± 6.59	91.01 ± 18.67	77.46 ± 8.31 *	95.11 ± 11.36
	10	93.83 ± 11.64 ^‡^	100.9 ± 23.33 ^§,‡^	70.27 ± 12.44 ^†^	100 ± 0 ^#,†,^*	96.05 ± 11.22	100.26 ± 22.25	78.53 ± 10.87 *	84.4 ± 11.6
	15	93.07 ± 18.78 ^‡^	93.26 ± 19.8 ^‡^	65.67 ± 8.03 ^†,^*	90.04 ± 11.91 ^#,^*	92.48 ± 15.22	97.1 ± 19.31	74.91 ± 8.27 *	87.24 ± 15.98
	20	94.62 ± 17.53 ^‡^	90.57 ± 16.4 ^‡^	65.99 ± 12.45 ^†,^*	77.38 ± 10.57 ^#^	92.71 ± 14.94	93.9 ± 15.96	75.74 ± 12.09 *	88.81 ± 11.9
	30	102.71 ± 9.75 ^§,‡^	87.04 ± 10.41 ^‡^	67.54 ± 13.38 ^#,†,^*	80.14 ± 16.56 ^#,^*	100.09 ± 8.86 ^§,‡^	88.8 ± 10.9	78.39 ± 11.44 ^#,^*	82.99 ± 14.03 ^#,^*
	40	95.56 ± 15.4 ^‡^	89.24 ± 15 ^‡^	64.64 ± 11.14 ^†,^*	81.29 ± 11.47 ^#^	94.16 ± 12.76	91.88 ± 15.07	74.95 ± 10.53 *	86.92 ± 16.4
	50	93.57 ± 11.8	89.88 ± 12.67	68.8 ± 12.93 *	76.21 ± 15.19	93.84 ± 9.81	91.82 ± 12.58	78.45 ± 11.99 *	89.15 ± 19.6
	60	93.58 ± 16.08	93.63 ± 16.39	77.35 ± 15.43 *	81.8 ± 18.3	94.78 ± 14.67	95.25 ± 15.94	86.35 ± 13.87 *	88.72 ± 12.89
	70	89.94 ± 10.71	97.37 ± 20.15	72.54 ± 15.35 *	83.61 ± 22.63	91.76 ± 10.43	97.93 ± 16.94	81.15 ± 12.41 *	87.45 ± 14.99
	80	95.14 ± 13.07	89.71 ± 14.84	76.92 ± 20.24 *	83.49 ± 15.35	95.46 ± 9.97	92.18 ± 14.1	84.42 ± 18.14 *	92.17 ± 15.59
	90	87.99 ± 8.46^‡^	88.58 ± 10.34 ^‡^	70.97 ± 10.36 ^†,^*	81.67 ± 17.32 ^#,‡^	88.79 ± 8.1	90.78 ± 9.33	79.97 ± 10.03 *	94.8 ± 16.15
	100	87.86 ± 9.3	88.64 ± 11.42	71.48 ± 13.18 *	87.56 ± 18.38	90.96 ± 9.59	91.16 ± 13.57	81.35 ± 11.03 *	95.16 ± 17.29
	110	83.45 ± 8.78	97.85 ± 20.7	74.6 ± 15.34 *	90.13 ± 17.9	86.76 ± 9.23	99.26 ± 18.36	83.22 ± 13.37 *	93.83 ± 18.07
	120	81.58 ± 11.22	92.77 ± 10.13	78.06 ± 10.19 *	91.04 ± 18.56	83.33 ± 8.68	93.89 ± 7.92	85.27 ± 7.98 *	93.54 ± 17.44
	130	92.99 ± 8.56	100.07 ± 21.17	79.95 ± 16.72 *	90.46 ± 21.32	93.26 ± 7.84	100.1 ± 18.69	87.33 ± 14.46 *	93 ± 15.23
	140	88.44 ± 11.76	98.27 ± 18.26	80.23 ± 13.23 *	90.44 ± 20.43	90.42 ± 10.46	98.82 ± 15.51	87.64 ± 12.37	86.23 ± 12.84
	150	91.75 ± 10.08	93.17 ± 13.38	82.31 ± 11.96 *	89.52 ± 18.93	94.38 ± 10.08	94.43 ± 11.32	88.56 ± 11.35	95.13 ± 17.5
	160	91.74 ± 10.02	92.35 ± 16.76	86.64 ± 18.84	82.6 ± 14.2	93.66 ± 7.92	94.19 ± 14.57	91.81 ± 15.59	90.05 ± 16.29
	170	86.56 ± 12.16	94.82 ± 17.22	87.05 ± 16.81	92.63 ± 22.64	87.9 ± 9.57	96.56 ± 16.65	92.18 ± 14.21	93.3 ± 12.6
	180	96.38 ± 9.87	90.06 ± 14.95	87.58 ± 17.12	86.06 ± 15.29	94.3 ± 8.25	91.74 ± 12.59	92.9 ± 15.52	98.03 ± 20.54

TP = total power, FA = force of acceleration. All variables are expressed as a mean percentage ± SD, relative to baseline values. ^#^ For a given variable, the value is significantly (*p* < 0.05) different from saline solution at that time point. ^§^ For a given variable, the value is significantly (*p* < 0.05) different from the combination of morphine and acepromazine value at that time point. ^‡^ For a given variable, the value is significantly (*p* < 0.05) different from the acepromazine value at that time point. ^†^ For a given variable, the value is significantly (*p* < 0.05) different from the morphine value at that time point. * For a given variable, the value is significantly (*p* < 0.05) different from the baseline value at that time point.

**Table 5:** Values at walk of energetic variables at −10, 5, 10, 15, 20, and then every 10 min (total period of 3 h) after IV injection (at 0 min) of 10 mL of sodium chloride (0.9%) solution (SS), 0.2 mg/kg of morphine hydrochloride solution diluted in sodium chloride (0.9%) to a volume of 10 mL (MOR), 0.02 mg/kg of acepromazine maleate diluted in sodium chloride (0.9%) to a volume of 10 mL (ACE), or a combination solution of 0.2 mg/kg of morphine hydrochloride and 0.02 mg/kg of acepromazine maleate diluted in sodium chloride (0.9%) to a volume of 10 mL (MOR + ACE).

VARIABLE		%DVP				%PP				%MLP			
TREATMENT		SS	MOR	ACE	MOR + ACE	SS	MOR	ACE	MOR + ACE	SS	MOR	ACE	MOR + ACE
Baseline		29.66 ± 5.11	30.42 ± 6.94	31.66 ± 7.21	31.57 ± 7.33	35.59 ± 7.58	36.17 ± 7.06	34.81 ± 7.61	35.12 ± 5.95	34.75 ± 6.03	33.41 ± 9.75	33.52 ± 6.85	33.31 ± 9.55
**Time after** **adminis-tration of treatment (min)**	5	28.11 ± 4.64	32.54 ± 4.2	26.72 ± 6.85 *	28.44 ± 4.64	36.12 ± 6.91	31.66 ± 3.83	34.51 ± 6.61	32.75 ± 0.73 *	35.77 ± 6.46	35.8 ± 2.54	38.76 ± 7.37	38.81 ± 4.67
	10	28.61 ± 4.77	34.37 ± 5.36 ^§,‡^	25.55 ± 6.26 ^†,^*	26.54 ± 2.62 ^†^	36.49 ± 6.33	31.04 ± 4.56	34.88 ± 5.93 *	33.97 ± 3.22 *	34.9 ± 7.14	34.59 ± 3.79	39.57 ± 7.11	39.49 ± 5.4
	15	29.66 ± 4.34	33.35 ± 5.11 ^§,‡^	24.75 ± 6.03 ^†,^*	27.22 ± 3.32 ^†^	35.65 ± 6.88 ^‡^	32.07 ± 4.4 ^‡^	33.48 ± 5.47 ^#,†,^*	34.39 ± 4.82	34.69 ± 5.77	34.58 ± 4.79	41.77 ± 5.89	38.39 ± 5.28
	20	32.61 ± 10.52 ^‡^	33.98 ± 6.38 ^‡^	24.02 ± 5.68 ^#,†,^*	28.2 ± 5.17	41.12 ± 15.09	31.01 ± 2.88	34.5 ± 6.37 *	33.63 ± 3.97	36.83 ± 8.21	35.01 ± 6.55	41.48 ± 7.03	38.18 ± 5.92
	30	29.36 ± 4.58	32.18 ± 5.72	23.98 ± 5.68 *	27.45 ± 4.48	35.46 ± 6.7	34.4 ± 6.1	34.63 ± 6.25 *	34.33 ± 3.84	35.18 ± 6.02	33.42 ± 6.79	41.39 ± 7.87	38.22 ± 6.22
	40	29.39 ± 3.4 ^‡^	31.23 ± 4.61 ^‡^	24.05 ± 5.03 ^#,§,†,^*	28.84 ± 3.88 ^‡^	35.05 ± 6.5	32.9 ± 5.14	33.7 ± 5.96 *	33.87 ± 4.39	35.28 ± 8.27	35.87 ± 5.43	42.25 ± 7.45	37.29 ± 4.88
	50	28.5 ± 2.59	32.09 ± 4.93	25.32 ± 6.5 *	25.93 ± 5.51 *	34.52 ± 6.45	34.71 ± 6.07	33.6 ± 5.88 *	33.8 ± 1.98 *	36.98 ± 6.6	33.21 ± 5.45	41.08 ± 7.44	40.27 ± 6
	60	28.62 ± 2.92	33.54 ± 5.21 ^§,‡^	25.54 ± 5.79 ^†,^*	28.18 ± 5.9 ^†^	35.38 ± 6.2	34.34 ± 5.6	33.89 ± 6.53 *	33.83 ± 4.46	36.01 ± 7.96	32.12 ± 5.64	40.57 ± 7.09	37.99 ± 8.2
	70	27.82 ± 4.81 ^†^	32.88 ± 4.08 ^#,§,‡^	24.74 ± 4.14 ^†,^*	27.82 ± 2.72 ^†^	34.46 ± 6.39	33.83 ± 6.41	35.37 ± 7.18 *	34.67 ± 3.64	37.72 ± 7.75	33.3 ± 3.93	39.89 ± 8.42	37.51 ± 2.76
	80	27.66 ± 5.62	31.54 ± 5.35	24.87 ± 4.63 *	29.46 ± 6.37	33.3 ± 6.55	35.16 ± 6.41	34.84 ± 5.97 *	34.36 ± 4.16	39.04 ± 7.56	33.31 ± 4.84	40.3 ± 7.08	36.18 ± 6.86
	90	27.64 ± 4.9	31.37 ± 4.28	24.82 ± 6.03 *	28.91 ± 6.08	34.37 ± 5.11	35.95 ± 6.8	34.67 ± 5.49 *	33.69 ± 3.41	37.99 ± 6.84	32.68 ± 6.12	40.5 ± 6.57	37.4 ± 6.22
	100	27.55 ± 5.32	31.3 ± 5.23	24.56 ± 5.36 *	27.08 ± 5.98	35.62 ± 6.69	36.08 ± 6.57	34.32 ± 6.29 *	34.27 ± 3.21	36.82 ± 8.78	32.62 ± 5.5	41.12 ± 7.76	38.65 ± 6.79
	110	26.94 ± 3.73	31.92 ± 5.22	25.31 ± 5.82 *	27.7 ± 4.3	35.42 ± 6.16	34.73 ± 7.14	34.07 ± 6.75 *	34.79 ± 2.94	37.64 ± 6.7	33.35 ± 5.86	40.62 ± 6.97	37.51 ± 6.71
	120	25.64 ± 4.3	30.5 ± 7.18	25.41 ± 5.22 *	27.6 ± 3.41	36.92 ± 5.79	35.46 ± 6.89	34.51 ± 6.87 *	35.01 ± 3.15	37.44 ± 7.56	34.05 ± 8.35	40.08 ± 6.09	37.39 ± 4.38
	130	27.27 ± 2.55	31.37 ± 5.3	26.01 ± 4.55 *	26.72 ± 2.86	34.42 ± 6.75	35.17 ± 8.18	35.8 ± 6.97 *	35.82 ± 3.81	38.31 ± 6.3	33.46 ± 7.76	38.19 ± 7.55	37.47 ± 4.11
	140	27.34 ± 4.83	32 ± 5.2	26.39 ± 5.41 *	27.34 ± 3.77	35.25 ± 8.65	34.24 ± 6.55	35.47 ± 6.67	37 ± 4.76	39.11 ± 7.12	33.76 ± 5.62	38.14 ± 6.27	35.66 ± 5.66
	150	27.4 ± 3.32	31.36 ± 5.67	26.61 ± 5.98 *	27.09 ± 2.8	35.37 ± 7.27	35.74 ± 6.63	34.3 ± 7.34	35.56 ± 4.82	38.08 ± 6.59	32.89 ± 6.56	39.09 ± 5.65	37.35 ± 4.14
	160	26.04 ± 3.27	30.01 ± 5.62	26.79 ± 5.44 *	27.7 ± 3.1	35.86 ± 6.05	34.79 ± 6.57	35 ± 8.16 *	36.99 ± 4.38	38.11 ± 6.83	35.2 ± 6.12	38.21 ± 8.14	35.31 ± 5.46
	170	25.38 ± 3.45	29.06 ± 4.95	27.04 ± 5.46 *	27.9 ± 5.14	33.97 ± 6.22	34.28 ± 6.28	33.8 ± 7.83	35.74 ± 4.73	40.65 ± 8.12	36.66 ± 5.4	39.16 ± 8.14	36.36 ± 6.37
	180	27.66 ± 2.88	30.02 ± 4.88	27.3 ± 6.97 *	28.21 ± 3.8	36.39 ± 6.45	36.6 ± 7.33	34.78 ± 8.46	35.62 ± 3.96	35.95 ± 7.82	33.38 ± 6.4	37.92 ± 7.89	36.17 ± 4.26

%DVP = dorsoventral component of the power, %PP = propulsion component of the power, %MLP = mediolateral component of the power. All variables are expressed as a mean percentage ± SD, relative to baseline values. ^#^ For a given variable, the value is significantly (*p* < 0.05) different from saline solution at that time point. ^§^ For a given variable, the value is significantly (*p* < 0.05) different from the combination of morphine and acepromazine value at that time point. ^‡^ For a given variable, the value is significantly (*p* < 0.05) different from the acepromazine value at that time point. ^†^ For a given variable, the value is significantly (*p* < 0.05) different from the morphine value at that time point. * For a given variable, the value is significantly (*p* < 0.05) different from the baseline value at that time point.

**Table 6:** Values at walk of tranquilization variable at −10, 5, 10, 15, 20, and then every 10 min (total period of 3 h) after IV injection (at 0 min) of 10 mL of sodium chloride (0.9%) solution (SS), 0.2 mg/kg of morphine hydrochloride solution diluted in sodium chloride (0.9%) to a volume of 10 mL (MOR), 0.02 mg/kg of acepromazine maleate diluted in sodium chloride (0.9%) to a volume of 10 mL (ACE), or a combination solution of 0.2 mg/kg of morphine hydrochloride and 0.02 mg/kg of acepromazine maleate diluted in sodium chloride (0.9%) to a volume of 10 mL (MOR + ACE).

VARIABLE		GLD			
TREATMENT		SS	MOR	ACE	MOR + ACE
Baseline		100 ± 0	100 ± 0	100 ± 0	100 ± 0
**Time after administration of treatment (min)**	5	102 ± 6.2	98 ± 8.53	90.45 ± 6.92	94.46 ± 6.41
	10	96.67 ± 7.31	100.33 ± 4.63	91.48 ± 6.27	99 ± 13.19
	15	100.83 ± 2.86	98 ± 5.18	85.27 ± 7.2	95.27 ± 11.27
	20	104.67 ± 6.83	98.83 ± 6.18	84.44 ± 7.9	91.91 ± 4.73
	30	103 ± 6.07	95.83 ± 8.18	81.69 ± 4.28	95.99 ± 7.69
	40	101.83 ± 10.74	94.67 ± 6.35	88.09 ± 9.52	93.13 ± 11.33
	50	104.17 ± 5.88	100 ± 6.99	85.21 ± 8.26	92.96 ± 8.89
	60	102.17 ± 9.06	101 ± 7.72	87.05 ± 8.16	93.43 ± 6.67
	70	100.17 ± 10.23	93 ± 8.37	84.82 ± 8.8	92.77 ± 6.39
	80	105.33 ± 9.29	95.33 ± 9.75	85.35 ± 9.52	94.5 ± 3.97
	90	96.17 ± 10.93	92 ± 7.18	86.52 ± 6.68	89.87 ± 6.35
	100	102.17 ± 6.43	95.83 ± 4.67	81.86 ± 7.15	92.22 ± 9.42
	110	99.33 ± 6.62	94 ± 8.65	85.06 ± 13.4	91.81 ± 9.83
	120	102.83 ± 4.07	96.17 ± 5.31	87.12 ± 10.22	88.77 ± 6.05
	130	101 ± 4.52	95.67 ± 6.92	87.21 ± 11.16	91.72 ± 6.63
	140	102 ± 6.16	95 ± 3.74	85.09 ± 10.46	90.35 ± 7.16
	150	105.5 ± 6.02	95.17 ± 2.64	82.26 ± 9.29	91.29 ± 7.14
	160	105 ± 6	95.33 ± 5.65	84.81 ± 9.12	91.11 ± 3.14
	170	105 ± 5.93	95.33 ± 2.42	91.47 ± 10.42	94.18 ± 5.29
	180	100.83 ± 7.81	96.67 ± 3.78	90.71 ± 8.34	96.49 ± 6.09

GLD = ground to lip distance. All variables are expressed as a mean percentage ± SD.

## Data Availability

Not applicable.

## References

[1] Clutton R.E. (2010). Opioid analgesia in horses. Vet. Clin. Equine.

[2] McFadzean W.J.M., Love E.J. (2019). Perioperative pain management in horses. Equine Vet. Educ..

[3] Muir W.W., Muir W.W., Hubbell J.A.E. (2009). Anxiolytics, Nonopioid Sedative-Analgesics, and Opioid Analgesics. Equine Anesthesia. Monitoring and Emergency Therapy.

[4] Sanchez L.C., Robertson S.A. (2014). Pain control in horses: What do we really know?. Equine Vet. J..

[5] Bennett R.C., Steffey E.P. (2002). Use of opioids for pain and anesthetic management in horses. Vet. Clin. Equine.

[6] Muir W.W. (1981). Drugs used to produce standing chemical restraint in horses. Vet. Clin. N. Am. Large Anim. Pract..

[7] Love E.J., Taylor P.M., Murrell J., Whay H.R. (2012). Effects of acepromazine, butorphanol and buprenorphine on thermal and mechanical nociceptive thresholds in horses. Equine Vet. J..

[8] Carregaro A.B., Ueda G.I., Censoni J.B., Bisetto S.P., Alonso B.B., Reginato G.M. (2020). Effect of methadone combined with acepromazine or detomidine on sedation and dissociative anesthesia in healthy horses. J. Equine Vet. Sci..

[9] Taylor P., Coumbe K., Henson F., Scott D., Taylor A. (2014). Evaluation of sedation for standing clinical procedures in horses using detomidine combined with buprenorphine. Vet. Anaesth. Analg..

[10] Poller C., Hopster K., Rohn K., Kästner S.B. (2013). Nociceptive thermal threshold testing in horses—Effect of neuroleptic sedation and neuroleptanalgesia at different stimulation sites. BMC Vet. Res..

[11] Lopes C., Luna S.P.L., Rosa A.C., Quarterone C., Crosignani N., Taylor P.M., Pantoja J.C., Puoli J.N.P. (2016). Antinociceptive effects of methadone combined with detomidine or acepromazine in horses. Equine Vet. J..

[12] Pascoe P.J., Taylor P.M. (2003). Effects of dopamine antagonists on alfentanil-induced locomotor activity in horses. Vet. Anaesth. Analg..

[13] Combie J., Dougherty J., Nugent E., Tobin T. (1979). The pharmacology of narcotic analgesics in the horse. IV. Dose and time response relationships for behavioral responses to morphine. meperidine, pentazocine, anileridine, methadone, and hydromorphone. J. Equine Med. Surg..

[14] López-Sanromán F.J., Montes Freilich G., Gomez-Cisneros D., Varela M., Santiago I., Manso-Díaz G. (2021). Accelerometric Evaluation of the Locomotor Pattern After Administration of Morphine in Conscious Healthy Horses. J. Equine Vet. Sci..

[15] López-Sanromán F.J., Holmbak-Petersen R., Santiago I., Gómez de Segura I.A., Barrey E. (2012). Gait analysis using 3D accelerometry in horses sedated with xylazine. Vet. J..

[16] Barrey E., Auvinet B., Couroucé A. (1995). Gait evaluation of race trotters using an accelerometric device. Equine Vet. J. Suppl..

[17] Leleu C., Bariller F., Cotrel C., Barrey E. (2004). Reproducibility of a locomotor test for trotter horses. Vet. J..

[18] Barrey E., Evans S.E., Evans D.L., Curtis R.A., Quinton R., Rose R.J. (2001). Locomotion evaluation for racing in thoroughbreds. Equine Vet. J. Suppl..

[19] Auvinet B., Berrut G., Touzard C., Moutel L., Collet N., Chaleil D., Barrey E. (2002). Reference data for normal subjects obtained with an accelerometric device. Gait Posture.

[20] Leleu C., Cotrel C., Barrey E. (2005). Relationships between biomechanical variables and race performance in french Standardbred trotters. Livest. Prod. Sci..

[21] Barthélémy I., Barrey E., Thibaud J.L., Uriarte A., Voit T., Blot S., Hogrel J.Y. (2009). Gait analysis using accelerometry in dystrophin-deficient dogs. Neuromuscul. Disord..

[22] López-Sanromán F.J., Gomez-Cisneros D., Varela del Arco M., Santiago Llorente I., Santos González M. (2015). The use of low doses of acepromazine as an aid for lameness diagnosis in horses: An accelerometric evaluation. Vet. Comp. Orthop. Traumatol..

[23] Tobin T., Ballard S. (1979). Pharmacology Review: The Phenotiazine “Tranquilizers”. J. Equine Med. Surg..

[24] Lerche P., Muir W.W., Muir W.W., Hubbell J.A.E. (2009). Perioperative Pain Management. Equine Anesthesia. Monitoring and Emergency Therapy.

[25] Weishaupt M.A., Hogg H.P., Auer J.A., Wiestner T. (2010). Velocitydependent changes of time, force and spatial parameters in Warmblood horses walking and trotting on a treadmill. Equine Vet. J. Suppl..

[26] López-Sanromán F.J., Holmbak-Petersen R., Varela M., del Alamo A.M., Santiago I. (2013). Accelerometric comparison of the locomotor pattern of horses sedated with xylazine hydrochloride, detomidine hydrochloride, or romifidine hydrochloride. Am. J. Vet. Res..

[27] Frigerio M., Gómez Cisneros D., Santiago Llorente I., Manso-Díaz G., López-Sanromán J. (2019). A kinematic comparison of the locomotor pattern of horses sedated with detomidine alone and in combination with low doses of butorphanol. Equine Vet. J..

[28] Ledoux I., Decostre V., Canal A., Hogrel J.Y. (2009). Relation between total power estimated from 3D accelerometric data and distance covered during the six-minute walk test. Neuromuscul. Dis..

[29] Keegan K.G., MacAllister C.G., Wilson D.A., Gedon C.A., Kramer J., Yonezawa Y., Maki H., Pai P.F. (2012). Comparison of an inertial sensor system with a stationary force plate for evaluation of horses with bilateral forelimb lameness. Am. J. Vet. Res..

[30] Cruz F.S.F., Carregaro A.B., Machado M., Antonow R.R. (2011). Sedative and cardiopulmonary effects of buprenorphine and xylazine in horses. Can. J. Vet. Res..

[31] Nolan A.M., Hall L.W. (1984). Combined use of sedatives and opiates in horses. Vet. Rec..

[32] Sanchez L.C., Elfenbein J.R., Robertson S.A. (2008). Effect of acepromazine, butorphanol, or N-butylscopolammonium bromide on visceral and somatic nociception and duodenal motility in conscious horses. Am. J. Vet. Res..

